# Clinical Effect of IRT-5 Probiotics on Immune Modulation of Autoimmunity or Alloimmunity in the Eye

**DOI:** 10.3390/nu9111166

**Published:** 2017-10-25

**Authors:** Jaeyoung Kim, Se Hyun Choi, Yu Jeong Kim, Hyun Jeong Jeong, Jin Suk Ryu, Hyun Ju Lee, Tae Wan Kim, Sin-Hyeog Im, Joo Youn Oh, Mee Kum Kim

**Affiliations:** 1 Laboratory of Ocular Regenerative Medicine and Immunology, Seoul Artificial Eye Center, Seoul National University Hospital Biomedical Research Institute, Seoul 03080, Korea; scullism81@gmail.com (J.K.); choisehyun88@naver.com (S.H.C.); 21cnasa@naver.com (Y.J.K.); hj-2024@hanmail.net (H.J.J.); enter2357@naver.com (J.S.R.); dalmuly@empas.com (H.J.L.); bonzoo1@snu.ac.kr (J.Y.O.); 2 Department of Ophthalmology, Seoul National University College of Medicine, Seoul 03080, Korea; 3 Department of Ophthalmology, Seoul Metropolitan Government Seoul National University Boramae Medical Center, Seoul 07061, Korea; twkim93@medimail.co.kr; 4 Division of Integrative Biosciences and Biotechnology, Pohang University of Science and Technology, Pohang 37673, Korea; iimsh@postech.ac.kr; 5 Academy of Immunology and Microbiology, Institute for Basic Science, Pohang 37673, Korea

**Keywords:** autoimmunity, alloimmunity, cornea, dry eye, experimental autoimmune uveitis, IRT-5 probiotics, immunomodulatory effect, transplantation

## Abstract

Background: Although the relation of the gut microbiota to a development of autoimmune and inflammatory diseases has been investigated in various animal models, there are limited studies that evaluate the effect of probiotics in the autoimmune eye disease. Therefore, we aimed to investigate the effect of IRT-5 probiotics consisting of *Lactobacillus casei*, *Lactobacillus acidophilus*, *Lactobacillus reuteri*, *Bifidobacterium bifidum*, and *Streptococcus thermophilus* on the autoimmunity of uveitis and dry eye and alloimmunity of corneal transplantation. Methods: Experimental autoimmune uveitis was induced by subcutaneous immunization with interphotoreceptor-binding protein and intraperitoneal injection of pertussis toxin in C57BL/6 (B6) mice. For an autoimmune dry eye model, 12-weeks-old NOD.B10.*H2^b^* mice were used. Donor cornea of B6 mice was transplanted into BALB/C mice. IRT-5 probiotics or phosphate buffered saline (PBS) were administered for three weeks immediately after induction of uveitis or transplantation. The inflammation score of the retinal tissues, dry eye manifestations (corneal staining and tear secretion), and graft survival were measured in each model. The changes of T cells were evaluated in drainage lymph nodes using fluorescence-activated cell sorting. Results: Retinal histology score in IRT-5 group of uveitis was lower than that in PBS group (*p* = 0.045). Ocular staining score was lower (*p* < 0.0001) and tear secretion was higher (*p* < 0.0001) in the IRT-5 group of NOD.B10.*H2^b^* mice than that in the PBS group. However, the graft survival in the IRT-5 group was not different from those of PBS group. The percentage of regulatory T cells was increased in the IRT-5-treated dry eye models (*p* = 0.032). The percentage of CD8^+^IL-17^hi^ (*p* = 0.027) and CD8^+^ interferon gamma (IFNγ)^hi^ cells (*p* = 0.022) were significantly decreased in the IRT-5-treated uveitis models and the percentage of CD8^+^IFNγ^hi^ cells was markedly reduced (*p* = 0.036) in IRT-5-treated dry eye model. Conclusion: Our results suggest that administration of IRT-5 probiotics may modulate clinical manifestations of autoimmunity in the eye, but not on alloimmunity of corneal transplantation.

## 1. Introduction 

Advances in genomic sequencing in gut microbiota have revealed the possible relationship of human disease and immune homeostasis with the gut microbiota [1,2]. The contribution of the commensal or pathogenic microbiota of the gut to a development of autoimmune and inflammatory diseases has been investigated in various animal models of demyelinating disease, inflammatory bowel disease and autoimmune uveitis [3,4,5]. In addition, the gut microbiota is reported to act as a key player in balancing immune responses between regulatory T (Treg) cells and Th17 cells at mucosal surfaces [6,7].

Probiotics are live microorganisms that can provide beneficial effects on the host organism by competing with potential pathogens or gut commensals [8]. Probiotics can induce tolerance or modification of the immune system for the use of biotherapy [8,9]. Recent studies have shown the *Lactobacilli* and *Bifidobacterium*-specific beneficial effect in modifying inflammation in rheumatoid arthritis (RA), inflammatory bowel disease (IBD) and atopic dermatitis (AD) [10,11,12]. IRT-5 probiotic formulation has immune-modulatory effects in a model of experimental autoimmune myasthenia gravis (MG), colitis, and experimental autoimmune encephalomyelitis (EAE) models, by modifying T cell responses or Treg-Th17 homeostasis [13,14,15].

Autoimmune dry eye related to Sjögren’s syndrome, systemic lupus erythematosus (SLE) and rheumatoid arthritis are a chronic, devastating and incurable disease. Autoimmune uveitis is also a chronically deteriorating disease that disturbs visual acuity. If non-drug biotherapy can modify disease to render it less reactive, a steroid-related complication may be lessened. Emerging evidence suggest that gut microbiota may trigger autoimmunity in SLE, Sjögren’s syndrome and autoimmune uveitis with autoreactive T cells [16,17,18]. Therefore, the purpose of the study was to investigate whether IRT-5 probiotics could have an effect on autoimmunity of experimental uveitis or dry eye by modulating autoreactive T cells. We also tried to determine whether IRT-5 related reaction was specific to autoreactive T cells or non-specifically suppressed both autoreactive and alloreactive T cells in the corneal transplantation model.

## 2. Materials and Methods

### 2.1. Animals

All the animals were managed in accordance with the Association for Research in Vision and Ophthalmology statement for the Use of Animals in Ophthalmic and Vision Research. The study protocols were approved by the Institutional Animal Care and Use Committee of Seoul National University Biomedical Research Institute (IAUCUC No. 15-0124-C1A0 and IAUCUC No. 15-0136-C1A0).

Six-week-old female B6 (C57BL/6J) mice and BALB/C mice were purchased from Orient Bio Inc. (Seongnam, Korea). B6 mice were used for the experimental autoimmune uveitis (EAU) model and the B6-to-BALB/C model was used in corneal allo-transplantation. Breeding pairs of non-obese diabetic NOD.B10.*H2^b^* mice were purchased from Jackson Laboratories (Bar Harbor, ME, USA) for breeding, and male NOD.B10.*H2^b^* mice were used for the autoimmune dry eye model. All the mice were bred under a specific pathogen-free environment and maintained at 22 °C–24 °C, relative humidity 55% ± 5%, with free access to water and food at the Mouse Facility at Biomedical Research Institute of Seoul National University Hospital. All the mice were pre-treated with combined antibiotics (ampicillin, 1 g/L; vancomycin, 500 mg/L; and metronidazole, 1 g/L; all from Sigma-Aldrich, St. Louis, MO, USA) in drinking water for 5 days before an oral gavage of IRT-5 (treated group) or phosphate buffered saline(PBS, control) to promote settlement of IRT-5 probiotics in the intestine. 

### 2.2. Preparation of the Probiotic Mixtures of IRT-5

Probiotic IRT-5 (consisting of *L. casei*, *L. acidophilus*, *L. reuteri*, *B. bifidum*, and *S. thermophilus*) powder contains 2 × 10^8^ colony forming units (CFU)/g of each strain [13,14]. The total amounts of IRT-5 probiotics were 1 × 10^9^ CFU in 300 μL PBS per mice for an oral gavage feeding [13,14]. All the probiotic strains were kindly provided by Korea Yakult Co. (Giheung, Korea).

### 2.3. Induction and Treatment of EAU

EAU was induced in B6 mice using the method as previously described [19]. Briefly, after pretreatment of antibiotics, 250 μg of human interphotoreceptor retinoid-binding protein(IRBP) peptide 1–20, GPTHLFQPSLVLDMAKVLLD (20 mg/mL; Peptron, Daejeon, Korea), emulsified in complete Freund adjuvant (Sigma) which contains Mycobacterium tuberculosis (2.5 mg/mL; BD DifcoTM, Franklin Lakes, NJ, USA) were injected into a footpad. Concurrently, the mice received 0.7 μg Pertussis toxin (300 μL; Sigma-Aldrich, St. Louis, MO, USA) intraperitoneally. Immediately after immunization, either 1 × 10^9^ IRT-5 probiotics in 300 μL PBS (*n* = 11) or 300 μL PBS (*n* = 10) alone was gavaged orally once a day for 3 weeks. Unimmunized B6 mice were used for negative control (*n* = 6). After 3 weeks of the treatment, the mice were sacrificed for the assay.

### 2.4. IRT-5Treatment in Autoimmune Dry Eye Model

Twelve-week-old male NOD.B10.*H2^b^* mice were used as the autoimmune dry eye model (*n* = 31). Either 1 × 10^9^ IRT-5 probiotics in 300 μL PBS (*n* = 16) or 300 μL PBS (*n* = 15) alone was gavaged orally once a day for 3 weeks after pre-treatment of antibiotics. After 3 weeks of the treatment, the mice were sacrificed for the assay.

### 2.5. Corneal Transplantation and IRT-5Treatment

After pre-treatment of antibiotics, full-thickness penetrating orthotopic corneal grafts were transplanted as previously described [20,21] with a few modifications. Seven-week-old B6 mice (H-2^b^) were served as corneal donors, and BALB/c mice (H-2^d^) used as recipients (allografts; *n* = 22). Donor corneal buttons (2.5 mm-diameter) were transplanted in a 2.0 mm host trephined-bed and sutured in place with six to eight interrupted 10-0 nylon sutures (Ethicon, Somerville, NJ, USA). Either 1 × 10^9^ IRT-5 probiotics in 300 μL PBS (*n* = 14) or 300 μL PBS (*n* = 8) alone was gavaged orally once a day for 3 weeks in allografted mice. Syngeneic corneal grafts (BALB/c-to-BALB/c) were also performed as negative controls (*n* = 6). After the surgery, all recipients received antibiotic eye drops (levofloxacin, Cravit^®^, Santen Pharmaceutical Co., Ltd., Osaka, Japan) once a day from the day of surgery (postoperative day, POD 0) for 14 days. All grafts were evaluated twice per week for 3 weeks. Graft rejection, defined as complete loss of graft transparency as mentioned in the previous study [22] was evaluated in a blinded manner. After 3 weeks of the treatment, the mice were sacrificed for the assay. 

### 2.6. Evaluation of Clinical Manifestations in Autoimmune Dry Eye Model

Under anesthesia with zoletil and xylazine, phenol red-impregnated cotton threads (FCI Ophthalmics, Pembroke, MA, USA) were administered into the lateral canthus of mice for 60 s, and wetting of the thread was measured in millimeters to evaluate the amount of tear production. For corneal epithelial defect observation, one drop of 3% Lissamine Green B (Sigma-Aldrich) was applied to the lower lateral conjunctival sac because it has convenience not requiring cobalt light excitation [23] under anesthesia. Dye staining of the cornea was scored in a blinded manner as follows: score 0 for no punctuate staining; score 1 when less than one-third of the cornea was stained; score 2 when two-thirds or less was stained; and score 3 when more than two-thirds were stained. 

### 2.7. Histology and Histological Scoring of EAU

On day 21 after immunization, the mice were euthanized by cervical dislocation after being anesthetized with zoletil, according to the American Veterinary Medical Association Guidelines for the Euthanasia of Animals (2013 Edition), and the eyeballs were harvested for the histopathologic examination. Eyeballs were fixed in 10% formaldehyde and paraffin-embedded. Serial 4-μm-thick sections were sliced and stained with hematoxylin and eosin (H&E). The histologic features were observed, and pathology scores were graded in a blinded manner on a scale of 0 to 4 according to the criteria previously described by Caspi [24].

### 2.8. Histopathology of ExtraorbitalGland in Autoimmune Dry Eye Model

The extraorbital gland was excised and fixed in 10% formalin. The samples were sliced into 4-μm-thick sections and stained using H&E. Inflammatory foci score (>50 inflammatory cells/focus = 1, 25–50 inflammatory cells/focus = 0.5) was measured in a blinded manner. 

### 2.9. Flow Cytometry Analysis of T CellSubsets

Cervical lymph nodes were collected in mice of uveitis (*n* = 3–6) and transplantation model (*n* = 6–11), and submandibular lymph nodes (*n* = 5) were collected in the mice of autoimmune dry eye model. The proportions of Th1, Th17, or Treg cells were determined by measuring IFN-γ, IL-17, or CD25 and Foxp3-expressing CD4^+^T cells using flow cytometry. To collect cell suspensions, lymph nodes were placed and minced between the frosted ends of two glass slides in RPMI media containing 10% fetal bovine serum (FBS) and 1% penicillin-streptomycin. The cells were immunostained using the following fluorescence-conjugated anti-mouse antibodies: CD4, CD25, Foxp3, IFNγ, major histocompatibility complex (MHC) II, CD11b, Ly6G and Ly6C (all from eBioscience, San Diego, CA, USA) and interleukin (IL)-17A (BD Pharmingen™, San Diego, CA, USA). For intracellular staining, the cells were stimulated for 4 h with 50 ng/mL phorbolmyristate acetate and 1 μg/mL ionomycin in the presence of GolgiPlug (BD Pharmingen™). The fluorescence assays of the cells were performed using a FACSCanto flow cytometer (BD BioSciences, Mountain View, CA, USA). The gate was set on CD4^+^ cell population, and further analysis of surface or intracellular markers was done within this gate. Gating strategies are shown in Supplementary Figure S1. For the gating of Treg cells, the gate was set on CD4 cells, and further gated with CD25^hi^ and FoxP3^hi^ cells. For the gating of monocytic Myeloid-Derived Suppressor Cells (mMDSC), the gate was set on MHC^lo^CD11b^hi^ cell population, and further gated with Ly6G^lo^ and Ly6C^hi^ cells. Data were analyzed using the FlowJo program (Tree Star, Inc., Ashland, OR, USA).

### 2.10. Statistical Analysis

All statistical tests were performed using Prism software (GraphPad Prism, Inc., La Jolla, CA, USA). Data were analyzed by one-way analysis of variance (ANOVA) or Dunnett’s multiple comparisons tests to compare more than two groups. Independent *t*-test or nonparametric Mann–Whitney *U* test was used for a comparison of two groups. To compare the changes over time, data were analyzed by paired *t*-test or Wilcoxon matched-pairs signed rank test. The survival was compared using the Log–Rank test. The data are presented as the mean ± standard error (SE). Differences were considered significant at *p* < 0.05.

## 3. Results

### 3.1. IRT-5Treatment Prevents Development of EAU

**Figure 1:**
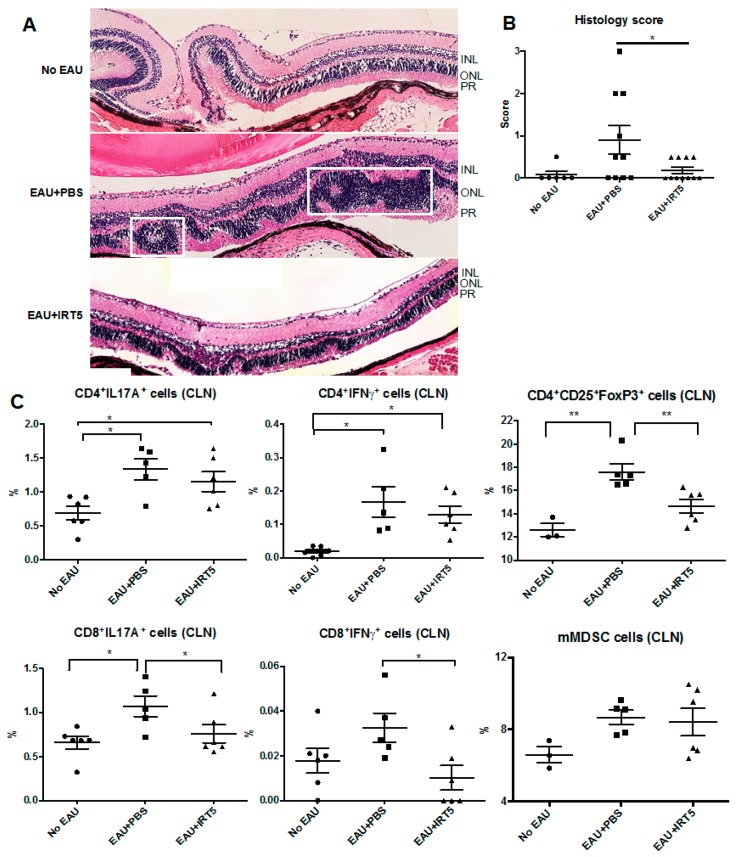
IRT-5 treatment suppresses inflammation of experimental autoimmune uveitis (EAU). (**A**) Hematoxylin and eosin (H&E) staining of cross-sections of retinal tissues. Disruption of photoreceptor layer and diffuse infiltration of inflammatory cells into the outer nuclear layer (boxes indicate inflammation foci) were remarkably reduced with few inflammatory cells in IRT-5-treated one. INL: inner nuclear layer, ONL: outer nuclear layer, PR: photoreceptor layer; (**B**) Histology score was lower in IRT-5-treated mice than in phosphate buffered saline(PBS)-treated mice (*p* = 0.045, one-way ANOVA and Dunnett’s multiple comparisons test); (**C**) Data depict the percentage of IFNγ or IL-17 secreting T cells, CD4^+^CD25^+^Foxp3^hi^T regulatory cells, and mMDSC among the total cervical lymph node(CLN) cells (* *p* < 0.05; ** *p* < 0.01; one-way ANOVA and Dunnett’s multiple comparisons test). The percentage of CD8^+^IL17^hi^ (*p* = 0.027) and CD8^+^IFNγ^hi^cells (*p* = 0.022) in IRT-5-treated mice were lower than in PBS-treated mice. The percentage of Treg cells was higher than in control (*p* = 0.0021), while it was lower in IRT-5-treated mice than in PBS-treated mice (*p* = 0.0021). Data are presented as mean ± standard errors.

We first evaluated whether daily oral gavage of IRT-5 could diminish inflammation in autoimmunity using EAU model of the B6 mouse. After 3 weeks treatment with IRT-5, disruption of photoreceptor layer was barely observed with few inflammatory cells. On the contrary, severe destruction of the photoreceptor layer was found with many inflammatory cells present in PBS-treated mice (Figure 1A). Histology score in IRT-5-treated mice was lower than in PBS-treated mice (*p* = 0.045, one-way ANOVA and Dunnett’s multiple comparisons test, Figure 1B). Flow cytometry of cervical lymph nodes showed that the percentage of CD8^+^IL17^hi^and CD8^+^IFNγ^hi^ cells in IRT-5-treated mice were lower than in PBS-treated mice (*p* = 0.027 and *p* = 0.022, respectively; one-way ANOVA and Dunnett’s multiple comparisons test). There were no significant differences regarding the percentage of IFNγ or IL-17 secreting CD4^+^ T cells and mMDSC. Unexpectedly, the percentage of Treg cells was higher in the uveitis model than in control (*p* = 0.0021), while the percentage of Treg cells was lower in IRT-5-treated mice than in PBS-treated mice (*p* = 0.0021, one-way ANOVA and Dunnett’s multiple comparisons test). This suggests that the modulation of autoreactive CD8^+^ T effector cells with IRT-5 seems not to be mediated by Treg cells in uveitis model. 

### 3.2. IRT-5 Treatment Attenuates Clinical Manifestations of Autoimmune Dry Eye Models

**Figure 2:**
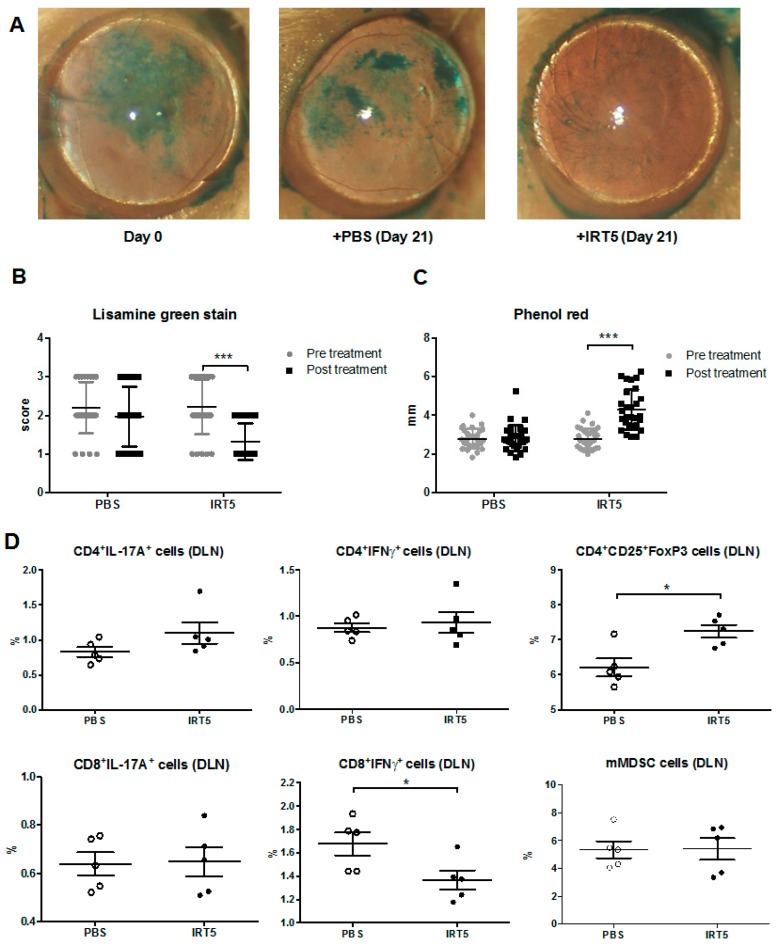
IRT-5 treatment reduces clinical manifestations of autoimmune dry eye model. (**A**) One set of the representative photos for the ocular staining scoring; (**B**) Ocular staining score was significantly decreased compared with the score of pretreated level in IRT-5-treated mice (*** *p* < 0.0001, paired *t*-test); (**C**) Phenol red thread test showed significant increase of tear secretion in IRT-5-treated mice(*** *p* < 0.0001, paired *t*-test); (**D**) Data depict the percentage of IFNγ or IL-17 secreting T cells, CD4^+^CD25^+^Foxp3^hi^ T regulatory cells, and mMDSC among the total draining lymph node(DLN) cells (* *p* < 0.05, Mann–Whitney *U* test). The percentage of CD8^+^IFNγ^hi^ cells in IRT-5-treated mice was lower than in PBS-treated mice (*p* = 0.036). The percentage of Treg cells was higher in IRT-5-treated mice were lower than in PBS-treated mice (*p* = 0.032); Data are presented as mean ± standard errors.

**Figure 3:**
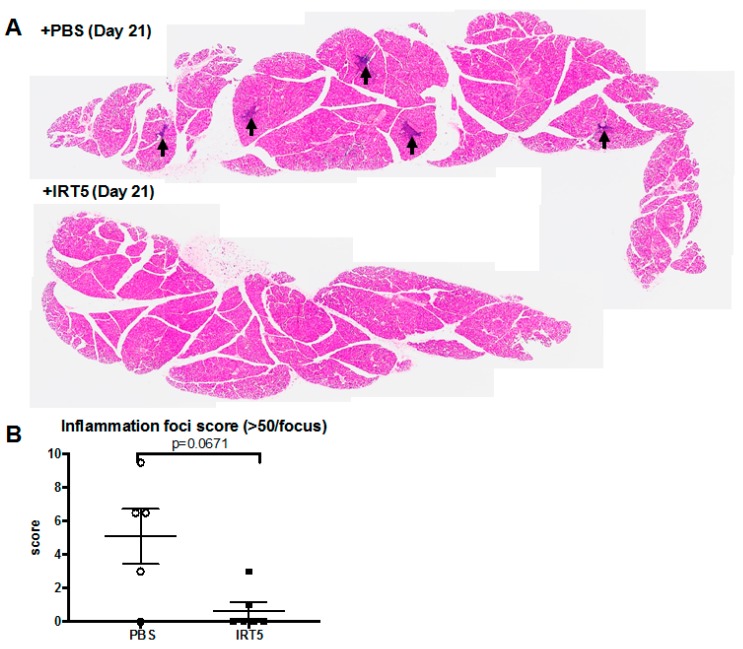
IRT-5 treatment attenuates inflammation of autoimmune dry eye models (**A**) One set of representative H&E cross-sections of extraorbital glands (upper panel; PBS-treated one, lower panel; IRT-5-treated one, arrows indicate inflammation foci which include more than 50 inflammatory cells); (**B**) Inflammation foci score is lower in IRT-5-treated mice than in PBS-treated mice with a marginal significance (*p* = 0.067, Mann–Whitney *U* test). Data are presented as mean ± standard errors.

In parallel, we assessed whether daily oral gavage of IRT-5 could be also effective in reduction of autoimmune dry eye manifestation using 12-week-old NOD.B10.*H2^b^* mice in the Sjögren’s disease model. Surprisingly, IRT-5 treatment reduced ocular staining (*p* < 0.0001, paired *t*-test) and increased tear secretion (*p* < 0.0001, paired *t*-test; Figure 2A–C). Flow cytometry of draining lymph nodes (submandibular LN) revealed that the percentage of CD8^+^IFNγ^hi^ cells in IRT-5-treated mice were lower than in PBS-treated mice (*p* = 0.036, Mann–Whitney U test), which was accompanied by the increased Treg cells in IRT-5-treated mice (*p* = 0.032, Mann–Whitney *U* test, Figure 2D). The percentage of mMDSC was not changed with IRT-5 treatment. H&E cross-sections of extraorbital glands show few inflammation foci in IRT-5-treated mice than in PBS-treated mice with a marginal significance (*p* = 0.067, Mann–Whitney *U* test; Figure 3A,B). It indicates that Treg cells may be involved in the induction of tolerance in autoimmune dry eye model with IRT-5 treatment. 

### 3.3. IRT-5Treatment Does Not Prolong the Survival of Corneal Allograft

**Figure 4:**
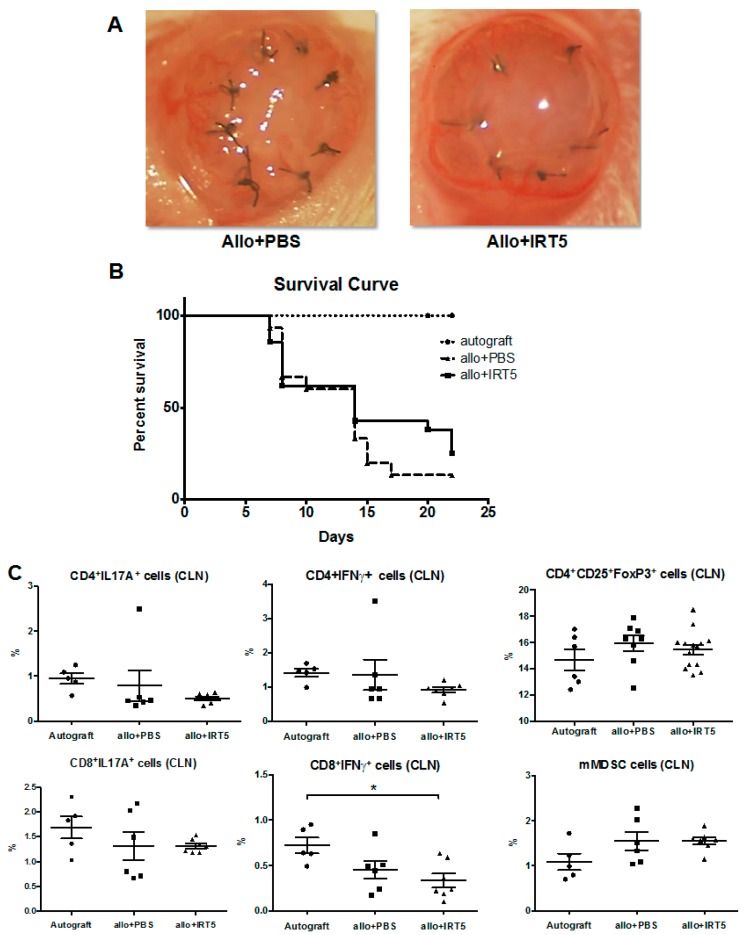
IRT-5 treatment does not prolong the survival of corneal allograft in B6-to BALB/C transplantation model. (**A**) Representative photographs of the cornea at day 22; (**B**) Survival curve of corneal allografts shows no statistical difference between in IRT-5-treated and PBS-treated mice (*p* = 0.3413, Log-Rank test); (**C**) Data depict the percentage of IFNγ or IL-17 secreting T cells, CD4^+^CD25^+^Foxp3^hi^Treg, and mMDSC among the total DLN cells (* *p* < 0.05, one-way ANOVA and Dunnett’s multiple comparisons test). There are no significant differences of T effector cells, Treg cells and mMDSC between in IRT-5-treated and PBS-treated mice.

Finally, we investigated whether the immunomodulatory effect of IRT-5 treatment could affect alloimmunity in B6-to BALB/C transplantation model. However, survival curve shows no prolongation of the corneal allografts in IRT-5-treated (*p* = 0.3413, Log-Rank test; Figure 4B). There were no significant changes of IFNγ or IL-17 secreting effector T cells, Treg cells, and mMDSC in cervical lymph nodes between IRT-5- and PBS-treated mice (Figure 4C). It suggests that IRT-5 treatment cannot suppress alloimmunity associated inflammation albeit it can suppress autoimmunity associated inflammation, which means treatment of IRT-5 is specific to autoimmunity. 

## 4. Discussion

Herein, we report that oral application of IRT-5 probiotics attenuates clinical disease of EAU and autoimmune dry eye by reducing autoreactive T cells. IRT-5 does not influence alloimmunity of corneal transplantation. As far as us aware of, this is the first report describing probiotics can be effective in attenuating autoimmune uveitis or autoimmune dry eye. The immune modulatory effect on ocular autoimmune disease well corresponds with the experimental outcome with IRT-5probiotics in the other autoimmune disease, such as MG, colitis, and EAE of previous reports [13,14,15]. The present data indicate that autoreactive T cells in EAU and dry eye may be cross-reactive to the gut antigen, while alloreactive T cells in corneal transplantation are not cross-reactive. Given that the severity of Sjögren’s syndrome is correlated with microbial dysbiosis [25], our study suggests a clinical relevance that IRT-5 may be beneficial in the patients with Sjögren’s syndrome. Since most of the study groups included less than 10 mice, further studies using larger numbers of animals would be required to clarify the results.

Probiotics have been investigated in clinical trials to prove their efficacy [26,27,28]. A meta-analysis regarding the effect of probiotics on the maintenance of remission in ulcerative colitis reported that relapse was lower in probiotic patients at one year than in placebo patients (Odds Ratio 0.27), suggesting a positive effect [28]. A double-blind, randomized, placebo-controlled trial with sixty children with perennial allergic rhinitis showed no additional benefit of *Lactobacillus paracasei* when used with regular levocetirizine, although there was a continuing decrease in symptomatic scores [27]. While, a prospective, open-label phase I/II controlled clinical trial with forty subjects with an ocular dysesthesia and comorbid enteral and anxiety-depression symptoms showed significant improvement of the symptoms in treated group with probiotic lysate as compared to the control group. This study suggests that probiotics may work on the ocular disease. Overall, the effect of probiotics appears to be dependent on the clinical disease entities or the applied microorganism strains. Through the emerge of the evidence, *Lactobacillus* and *Bifidobacterium* are known to be one of the key players in producing immune modulatory molecules or Treg cells [10,12,29]. However, the exact underlying mechanism of probiotics on human immune system remains unexplored. 

Regarding the IRT-5 probiotics used in this study, five strains exhibiting high secretion of IL-10 and low secretion of IL-12 in the ex vivo co-culture system with immune cells were selected by the collaborator (S.H. Im) [29,30]. The IRT-5 mixture includes both *Lactobacillus* and *Bifidobacterium*. The mode of action of IRT-5 probiotics is still unclear, and its efficacy seems to differ depending on the underlying autoimmune disease or recipient mouse strain as similar to other probiotics. Nevertheless, the mixture of multiple strains is regarded to be synergistic, with enhanced survival in the intestine compared with a single strain [29]. 

Understanding the pathogenesis of autoimmune ocular diseases, such as uveitis and Sjögren’s syndrome, is imperative to develop the therapeutic intervention. Etiologies of uveitis and dry eye are heterogeneous and there is no single animal model. However, the evidence is emerging that T cell-targeted therapy can be used to treat both diseases [31]. Th17 cells and IL-17 are known to be one of the key players in both diseases [5,32]. The role in CD8^+^T cells in autoimmune disease is debatable. One study reported CD8^+^T cells may attenuate dry eye by decreasing IL-17A producing cells in the B6 mouse model [33]. Another study described that autoimmunity can be also induced by antigen-specific CD8^+^T cells in the model of uveitis, albeit CD4^+^T cell-driven disease being dominant [34]. In this regard, it can be presumed that autoreactive CD8^+^ T cells may be cross-reactive to gut antigen in this uveitis model, and the decrease of CD8^+^IL17^hi^ and CD8^+^IFNγ^hi^ cells in IRT-5-treated mice of our study may contribute to the prevention of uveitis. Clinical symptoms in keratoconjunctivitis sicca in both Sjögren’s syndrome and non-Sjögren dry eye are also known to be dependent on T cell activation, both CD4^+^ and CD8^+^T cells [35,36,37]. In Sjögren’s syndrome, pathogenesis relating to microorganism are proposed as follows [38]; (1) autoreactive T cells against Ro-60 could be activated by the peptides from oral, skin and gut bacteria; (2) immature B cells may not be appropriately removed to increase autoreactive B cells by failing access to gut-associated lymphoid tissue; (3) Ro-60 activated T cells could activate autoreactive B cells; and (4) dysbiosis of the gut microbiota could increase Th17 cells to migrate systemic circulation. With this line of reasoning, it is conceivable that the IRT-5 probiotics might suppress cross-reactive T cells against gut peptides in this dry eye model, and therefore it might make the clinical manifestation improved. 

Considering that Th17 cells may be more effective than Th1 cells to promote B cell proliferating and differentiating in dry eye [32] or Th17 cells per se are pathogenic in inflammatory disease [7,37,39], it would be better to use the Th17-associated dry eye model. In fact, NOD.B10.*H2^b^* mice, whose autoreactive T cells contribute to pathogenesis, are available only from Jackson Laboratory. Because we purchased NOD.B10.*H2^b^* from this source, where most of the mice lack Th17 cells in their immune system compared with those from Taconic Laboratory, we could not see significant changes in IL-17 secreting T cells in our dry eye model unlike in the autoimmune uveitis model. Nevertheless, clinical expression of the disease was attenuated accompanied by an increase of Treg cells and a decrease of CD8^+^IFNγ^hi^ cells. In patients and in the NOD mouse model of Sjögren’s syndrome, lymphocytic infiltrates are known to consist of CD4 and CD8 T cells in the lacrimal glands. However, the role of CD8^+^ T cells in pathogenesis is still underexplored. A recent report showed that CD8^+^ T cells contribute to the pathogenesis of lacrimal gland autoimmunity [40]. Adoptive transfer of CD8^+^ T cells isolated from the NOD mice into NOD-SCID recipients resulted in inflammation of the lacrimal glands regardless of the presence of CD4 T cells [40]. Given these observations, it seems reasonable to hypothesize that the decrease of CD8^+^IFNγ^hi^ cells by IRT-5 may contribute to the improvement of clinical dry eye manifestation that we observed. How exactly to down-regulate effector T cells in each model is currently under investigation in our lab.

Gut microbiota appears to affect systemic disease in two different ways in animal models [17]. Type 1 diabetes in NOD mice is ameliorated by the presence of microbiota, and germ-free mice enhance disease [41]. De Paiva et al. also reported that 24 days of treatment with broad spectrum antibiotics tends to worsen dry eye with an increase of T cells [25]. On the contrary, the absence of microbiota or alteration in its composition can attenuate expression of autoimmune diseases. Experimental arthritis, EAE or EAU can be ameliorated in antibiotic-treated or germ-free mice [4,5,42]. Hori et al. showed that depletion of the gut microbiota by treatment with broad-spectrum antibiotics which had been given to pregnant dams and had continued after weaning (about 4 weeks) significantly attenuated uveitis of R161H mice. That is, dysbiosis of intestinal microbiota may differently effect on their autoimmunity depending on the disease entity or models. Considering that administration of antibiotics diminishes preexisting microbiota in the gut, pre-treatment of antibiotics in our study setting can be debated regarding the ascertain effect of antibiotics per se on disease expression. In fact, the purpose of pre-treatment was to reduce pre-existing microorganism partially enough to get a space for settling-down of the newly incoming IRT-5 commensals. However, we believe the pre-treatment effect would be not detrimental because the application time was very short (5 days, Supplementary Figure S2), and pre-treatment of antibiotics was done in both the control group and IRT-5-treated group to reduce the bias.

## 5. Conclusions

In summary, our study suggests that IRT-5 probiotics may have a benefit to attenuate clinical manifestation in EAU or autoimmune dry eye but not in alloimmunity of corneal transplantation.

## Supplementary Materials

The following are available online at www.mdpi.com/2072-6643/9/11/1166/s1, Figure S1: Gating strategies for IFNγ or IL-17 secreting T cells (A), CD4^+^CD25^+^Foxp3^hi^ T regulatory cells (Treg, B), and monocytic Myeloid-Derived Suppressor Cells (mMDSC, C), Figure S2: In the EAU model, there were no statistically significant differences in the percentage of IFNγ or IL-17 secreting T cells, and CD4^+^CD25^+^Foxp3^hi^ T regulatory cells among the total cervical lymph node (CLN) regardless of antibiotics pretreatment. AB stands for antibiotics pre-treatment.

## References

[1] Segre J.A. (2015). Microbiome. Microbial Growth Dynamics and Human Disease. Science.

[2] Cerf-Bensussan N., Gaboriau-Routhiau V. (2010). The Immune System and the Gut Microbiota: Friends or Foes?. Nat. Rev. Immunol..

[3] Chu H., Khosravi A., Kusumawardhani I.P., Kwon A.H., Vasconcelos A.C., Cunha L.D., Mayer A.E., Shen Y., Wu W.L., Kambal A. (2016). Gene-Microbiota Interactions Contribute to the Pathogenesis of Inflammatory Bowel Disease. Science.

[4] Berer K., Mues M., Koutrolos M., Rasbi Z.A., Boziki M., Johner C., Wekerle H., Krishnamoorthy G. (2011). Commensal Microbiota and Myelin Autoantigen Cooperate to Trigger Autoimmune Demyelination. Nature.

[5] Horai R., Zarate-Blades C.R., Dillenburg-Pilla P., Chen J., Kielczewski J.L., Silver P.B., Jittayasothorn Y., Chan C.C., Yamane H., Honda K. (2015). Microbiota-Dependent Activation of an Autoreactive T Cell Receptor Provokes Autoimmunity in an Immunologically Privileged Site. Immunity.

[6] Ohnmacht C., Park J.H., Cording S., Wing J.B., Atarashi K., Obata Y., Gaboriau-Routhiau V., Marques R., Dulauroy S., Fedoseeva M. (2015). Mucosal Immunology. The Microbiota Regulates Type 2 Immunity through Rorgammat(+) T Cells. Science.

[7] Sefik E., Geva-Zatorsky N., Oh S., Konnikova L., Zemmour D., McGuire A.M., Burzyn D., Ortiz-Lopez A., Lobera M., Yang J. (2015). Mucosal Immunology. Individual Intestinal Symbionts Induce a Distinct Population of Rorgamma(+) Regulatory T Cells. Science.

[8] Alok A., Singh I.D., Singh S., Kishore M., Jha P.C., Iqubal M.A. (2017). Probiotics: A New Era of Biotherapy. Adv. Biomed. Res..

[9] Pamer E.G. (2016). Resurrecting the Intestinal Microbiota to Combat Antibiotic-Resistant Pathogens. Science.

[10] So J.S., Kwon H.K., Lee C.G., Yi H.J., Park J.A., Lim S.Y., Hwang K.C., Jeon Y.H., Im S.H. (2008). Lactobacillus Casei Suppresses Experimental Arthritis by Down-Regulating T Helper 1 Effector Functions. Mol. Immunol..

[11] Viljanen M., Pohjavuori E., Haahtela T., Korpela R., Kuitunen M., Sarnesto A., Vaarala O., Savilahti E. (2005). Induction of Inflammation as a Possible Mechanism of Probiotic Effect in Atopic Eczema-Dermatitis Syndrome. J. Allergy Clin. Immunol..

[12] Kim N., Kunisawa J., Kweon M.N., Ji G.E., Kiyono H. (2007). Oral Feeding of Bifidobacterium Bifidum (Bgn4) Prevents Cd4(+) Cd45rb(High) T Cell-Mediated Inflammatory Bowel Disease by Inhibition of Disordered T Cell Activation. Clin. Immunol..

[13] Jeong J.J., Woo J.Y., Ahn Y.T., Shim J.H., Huh C.S., Im S.H., Han M.J., Kim D.H. (2015). The Probiotic Mixture Irt5 Ameliorates Age-Dependent Colitis in Rats. Int. Immunopharmacol..

[14] Chae C.S., Kwon H.K., Hwang J.S., Kim J.E., Im S.H. (2012). Prophylactic Effect of Probiotics on the Development of Experimental Autoimmune Myasthenia Gravis. PLoS ONE.

[15] Kwon H.K., Kim G.C., Kim Y., Hwang W., Jash A., Sahoo A., Kim J.E., Nam J.H., Im S.H. (2013). Amelioration of Experimental Autoimmune Encephalomyelitis by Probiotic Mixture Is Mediated by a Shift in T Helper Cell Immune Response. Clin. Immunol..

[16] Horai R., Sen H.N., Caspi R.R. (2017). Commensal Microbiota as a Potential Trigger of Autoimmune Uveitis. Expert Rev. Clin. Immunol..

[17] Zarate-Blades C.R., Horai R., Caspi R.R. (2016). Regulation of Autoimmunity by the Microbiome. DNA Cell Biol..

[18] Szymula A., Rosenthal J., Szczerba B.M., Bagavant H., Fu S.M., Deshmukh U.S. (2014). T Cell Epitope Mimicry between Sjogren’s Syndrome Antigen a (Ssa)/Ro60 and Oral, Gut, Skin and Vaginal Bacteria. Clin. Immunol..

[19] Lee H.J., Ko J.H., Jeong H.J., Ko A.Y., Kim M.K., Wee W.R., Yoon S.O., Oh J.Y. (2015). Mesenchymal Stem/Stromal Cells Protect against Autoimmunity Via Ccl2-Dependent Recruitment of Myeloid-Derived Suppressor Cells. J. Immunol..

[20] Niederkorn J.Y., Stevens C., Mellon J., Mayhew E. (2006). Cd4^+^ T-Cell-Independent Rejection of Corneal Allografts. Transplantation.

[21] Ko J.H., Lee H.J., Jeong H.J., Kim M.K., Wee W.R., Yoon S.O., Choi H., Prockop D.J., Oh J.Y. (2016). Mesenchymal Stem/Stromal Cells Precondition Lung Monocytes/Macrophages to Produce Tolerance against Allo- and Autoimmunity in the Eye. Proc. Natl. Acad. Sci. USA.

[22] Sonoda Y., Streilein J.W. (1993). Impaired Cell-Mediated Immunity in Mice Bearing Healthy Orthotopic Corneal Allografts. J. Immunol..

[23] Chen Y.T., Li S., Nikulina K., Porco T., Gallup M., McNamara N. (2009). Immune Profile of Squamous Metaplasia Development in Autoimmune Regulator-Deficient Dry Eye. Mol. Vis..

[24] Caspi R.R. (2003). Experimental Autoimmune Uveoretinitis in the Rat and Mouse. Curr. ProtocImmunol..

[25] De Paiva C.S., Jones D.B., Stern M.E., Bian F., Moore Q.L., Corbiere S., Streckfus C.F., Hutchinson D.S., Ajami N.J., Petrosino J.F. (2016). Altered Mucosal Microbiome Diversity and Disease Severity in Sjogren Syndrome. Sci. Rep..

[26] Feher J., Pinter E., Kovacs I., Helyes Z., Kemeny A., Markovics A., Plateroti R., Librando A., Cruciani F. (2014). Irritable Eye Syndrome: Neuroimmune Mechanisms and Benefits of Selected Nutrients. Ocul. Surf..

[27] Lin W.Y., Fu L.S., Lin H.K., Shen C.Y., Chen Y.J. (2014). Evaluation of the Effect of Lactobacillus Paracasei (Hf.A00232) in Children (6–13 Years Old) with Perennial Allergic Rhinitis: A 12-Week, Double-Blind, Randomized, Placebo-Controlled Study. Pediatr. Neonatol..

[28] Naidoo K., Gordon M., Fagbemi A.O., Thomas A.G., Akobeng A.K. (2011). Probiotics for Maintenance of Remission in Ulcerative Colitis. Cochrane Database Syst. Rev..

[29] Kang H.J., Im S.H. (2015). Probiotics as an Immune Modulator. J. Nutr. Sci. Vitaminol..

[30] Kwon H.K., Lee C.G., So J.S., Chae C.S., Hwang J.S., Sahoo A., Nam J.H., Rhee J.H., Hwang K.C., Im S.H. (2010). Generation of Regulatory Dendritic Cells and Cd4^+^Foxp3^+^ T Cells by Probiotics Administration Suppresses Immune Disorders. Proc. Natl. Acad. Sci. USA.

[31] Bose T., Diedrichs-Mohring M., Wildner G. (2016). Dry Eye Disease and Uveitis: A Closer Look at Immune Mechanisms in Animal Models of Two Ocular Autoimmune Diseases. Autoimmun. Rev..

[32] Subbarayal B., Chauhan S.K., di Zazzo A., Dana R. (2016). Il-17 Augments B Cell Activation in Ocular Surface Autoimmunity. J. Immunol..

[33] Zhang X., Schaumburg C.S., Coursey T.G., Siemasko K.F., Volpe E.A., Gandhi N.B., Li D.Q., Niederkorn J.Y., Stern M.E., Pflugfelder S.C. (2014). Cd8(+) Cells Regulate the T Helper-17 Response in an Experimental Murine Model of Sjogren Syndrome. Mucosal Immunol..

[34] Lee R.W., Nicholson L.B., Sen H.N., Chan C.C., Wei L., Nussenblatt R.B., Dick A.D. (2014). Autoimmune and Autoinflammatory Mechanisms in Uveitis. Semin. Immunopathol..

[35] Stern M.E., Gao J., Schwalb T.A., Ngo M., Tieu D.D., Chan C.C., Reis B.L., Whitcup S.M., Thompson D., Smith J.A. (2002). Conjunctival T-Cell Subpopulations in Sjogren’s and Non-Sjogren’s Patients with Dry Eye. Investig. Ophthalmol. Vis. Sci..

[36] Guzman M., Keitelman I., Sabbione F., Trevani A.S., Giordano M.N., Galletti J.G. (2016). Desiccating Stress-Induced Disruption of Ocular Surface Immune Tolerance Drives Dry Eye Disease. Clin. Exp. Immunol..

[37] Stern M.E., Schaumburg C.S., Pflugfelder S.C. (2013). Dry Eye as a Mucosal Autoimmune Disease. Int. Rev. Immunol..

[38] Van der Meulen T.A., Harmsen H., Bootsma H., Spijkervet F., Kroese F., Vissink A. (2016). The Microbiome-Systemic Diseases Connection. Oral Dis..

[39] Monteleone I., Sarra M., Pallone F., Monteleone G., Pilat N., Wekerle T., Ferrari-Lacraz S., Tiercy J.M., Villard J., Kerr D. (2012). Th17-Related Cytokines in Inflammatory Bowel Diseases: Friends or Foes?. Curr. Mol. Med..

[40] Barr J.Y., Wang X., Meyerholz D.K., Lieberman S.M. (2017). Cd8 T Cells Contribute to Lacrimal Gland Pathology in the Nonobese Diabetic Mouse Model of Sjogren Syndrome. Immunol. Cell Biol..

[41] Wen L., Ley R.E., Volchkov P.Y., Stranges P.B., Avanesyan L., Stonebraker A.C., Hu C., Wong F.S., Szot G.L., Bluestone J.A. (2008). Innate Immunity and Intestinal Microbiota in the Development of Type 1 Diabetes. Nature.

[42] Wu H.J., Ivanov I.I., Darce J., Hattori K., Shima T., Umesaki Y., Littman D.R., Benoist C., Mathis D. (2010). Gut-Residing Segmented Filamentous Bacteria Drive Autoimmune Arthritis Via T Helper 17 Cells. Immunity.

