# Supplementary material for: Clinical Effect of IRT-5 Probiotics on Immune Modulation of Autoimmunity or Alloimmunity in the Eye
Source: Nutrients. 2017 Oct 25;9(11):1166. doi: 10.3390/nu9111166 (PMC5707638; doi:10.3390/nu9111166)
Supplement: Supplementary file 1 [file nutrients-09-01166-s001.zip › nutrients-232832-supplementary.pdf]

nutrients

MDPI

Supplementary: Clinical Effect of IRT-5 Probiotics on Immune Modulation of Autoimmunity or Alloimmunity in the Eye

Jaeyoung Kim [1,2], Se Hyun Choi [1,2], Yu Jeong Kim [1,2], Hyun Jeong Jeong [1], Jin Suk Ryu [1], Hyun Ju Lee [1], Tae Wan Kim [3], Sin-Hyeog Im [4,5], Joo Youn Oh [1,2] and Mee Kum Kim [1,2,*]

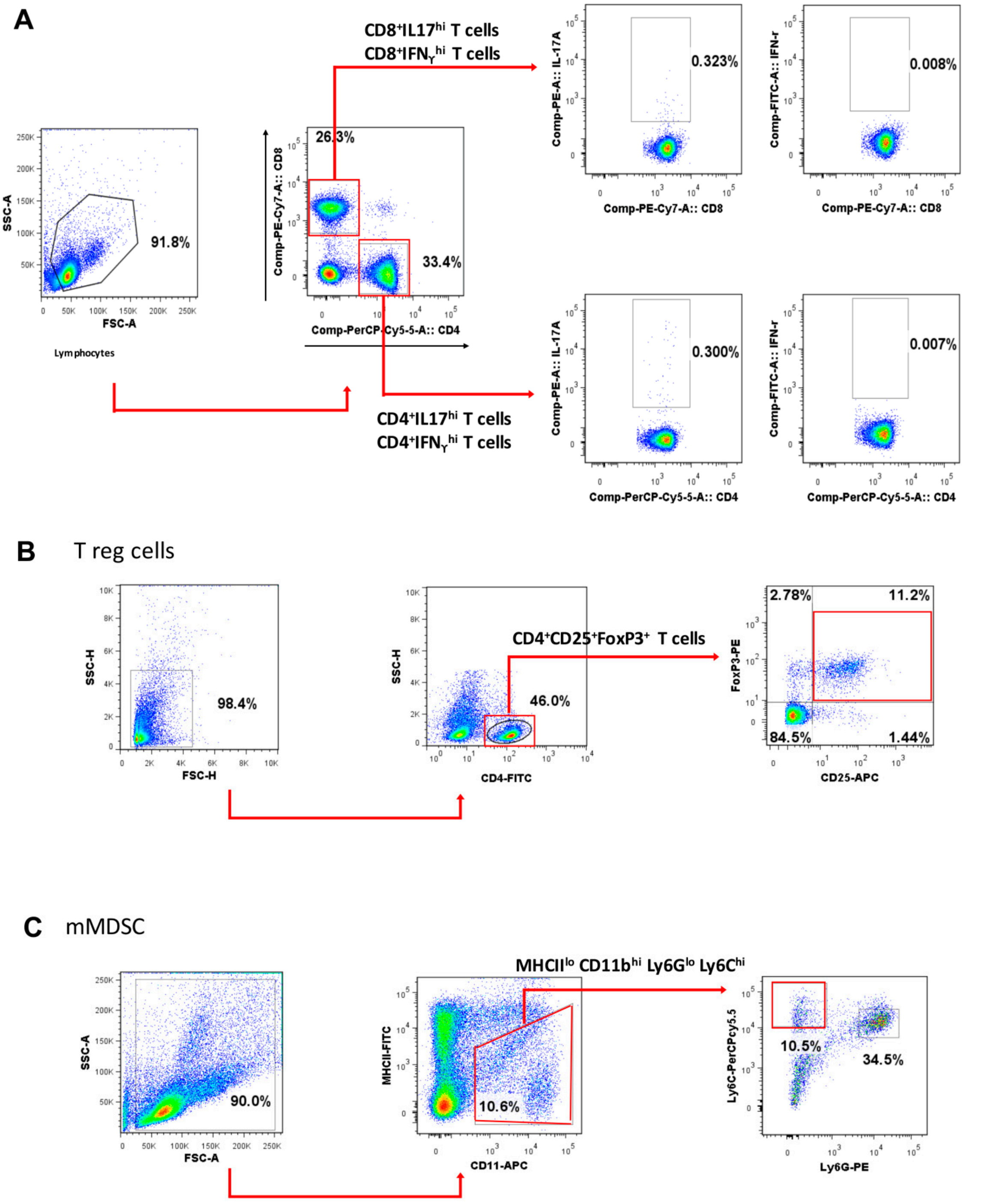

Figure S1. Gating strategies for IFNγ or IL-17 secreting T cells (**A**), $CD4^+CD25^+Foxp3^{hi}$ T regulatory cells (Treg, **B**), and monocytic Myeloid-Derived Suppressor Cells (mMDSC, **C**).

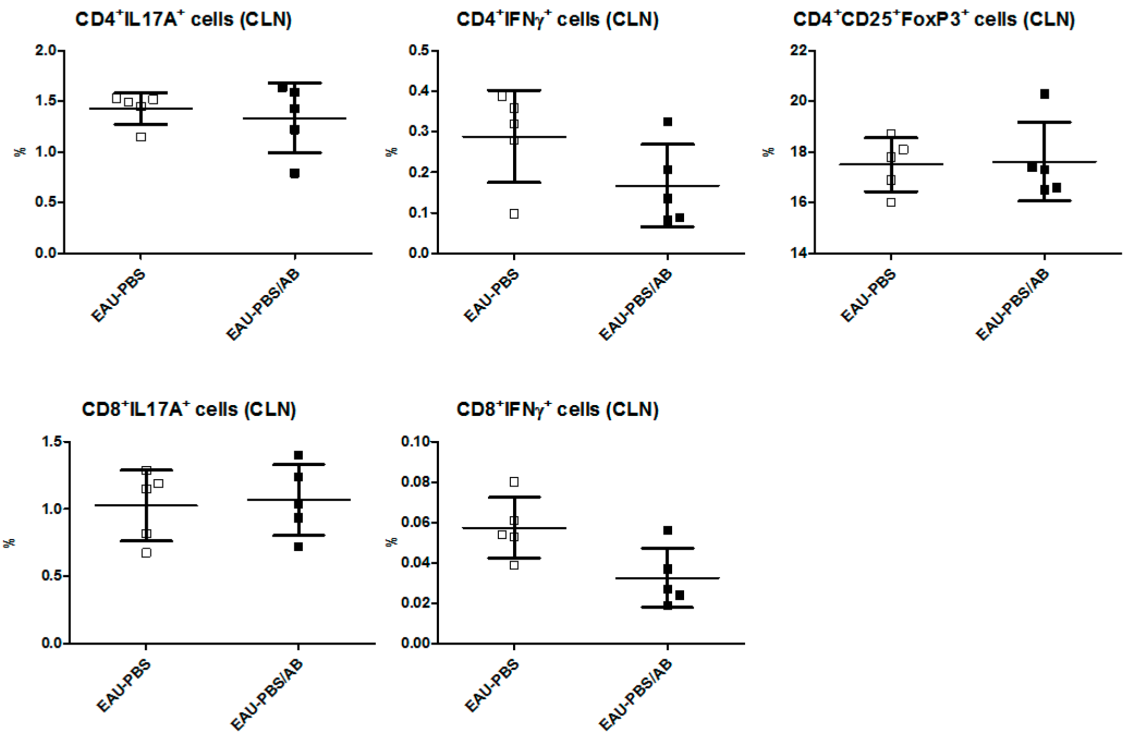

Figure S2. In the EAU model, there were no statistically significant differences in the percentage of IFNγ or IL-17 secreting T cells, and $CD4^+CD25^+Foxp3^{hi}$ T regulatory cells among the total cervical lymph node (CLN) regardless of antibiotics pretreatment. AB stands for antibiotics pre-treatment.
